# IL-1 Inhibitors in the Treatment of Familial Mediterranean Fever: Treatment Indications and Clinical Features in a Large Real-World Cohort

**DOI:** 10.3390/jcm13123375

**Published:** 2024-06-07

**Authors:** Melek Yalcin-Mutlu, Ozan Cemal Icacan, Fatih Yildirim, Selahattin Alp Temiz, Filippo Fagni, Georg Schett, Koray Tascilar, Ioanna Minopoulou, Gokhan Burul, Cemal Bes

**Affiliations:** 1 Department of Medicine 3—Rheumatology and Immunology, Friedrich-Alexander-Universität Erlangen-Nürnberg and Uniklinikum Erlangen, 91054 Erlangen, Germany; alp.temiz@uk-erlangen.de (S.A.T.); filippo.fagni@uk-erlangen.de (F.F.); georg.schett@uk-erlangen.de (G.S.); koray.tascilar@uk-erlangen.de (K.T.); ioanna.minopoulou@uk-erlangen.de (I.M.); 2 Deutsches Zentrum für Immuntherapie (DZI), Friedrich-Alexander-Universität Erlangen-Nürnberg and Uniklinikum Erlangen, 91054 Erlangen, Germany; 3 Department of Rheumatology, University of Health Sciences, Basaksehir Cam and Sakura City Hospital, Istanbul 34480, Türkiye; f08fatih@gmail.com (F.Y.); cemalbes@hotmail.com (C.B.); 4 Department of Rheumatology, Yozgat City Hospital, Yozgat 66100, Türkiye; ozancemalicacan@gmail.com; 5 Centre for Rare Diseases Erlangen, Friedrich-Alexander-Universität Erlangen-Nürnberg and Universitätsklinikum Erlangen, 91054 Erlangen, Germany; 6 Department of Internal Medicine, University of Health Sciences, Bagcilar Training and Research Hospital, Istanbul 34203, Türkiye; gokhanburul@hotmail.com

**Keywords:** familial mediterranean fever, colchicine resistance, colchicine intolerance, interleukin-1 inhibitors, amyloidosis

## Abstract

**Background:** The accruing evidence about the efficacy of anti-IL-1 agents in Familial Mediterranean Fever (FMF) patients led to their widespread off-label use. Therefore, identifying precise indications and clinical characteristics of IL-1i-warranting patients are important. This study investigated the clinical characteristics and treatment indications of patients with FMF requiring interleukin 1 inhibition therapy (IL-1i). **Methods:** Hospital records of FMF patients attending a tertiary care center at the Department of Rheumatology, University of Health Sciences, Basaksehir Cam and Sakura City Hospital were retrospectively analyzed. Data on symptoms and disease manifestations, age of symptom onset, time to diagnosis, MEFV variants, type of treatment, and their indications were collected. **Results:** Between June 2020 and March 2023, 312 FMF patients were identified. The mean age at the onset of symptoms was 14.0, and the mean time to diagnosis was 11.9 years. In total, 87.1% of patients were receiving colchicine monotherapy, while the remaining 11.8% warranted IL-1i. Clinical symptoms and flare manifestations did not show a significant difference between the two groups. However, patients receiving IL-1i started having symptoms at younger age (11.5 vs. 14.5, *p* = 0.042) and time to diagnosis was longer (18.2 vs. 11.0, *p* < 0.01). M694V homozygosity was more common in patients receiving IL-1i. Indications for patients receiving IL-1i were colchicine resistance (8.0%), secondary amyloidosis (5.1%), and colchicine intolerance (2.2%). **Conclusions:** This study shows that a subset of FMF patients, particularly those with a more severe phenotype with an earlier disease onset and M694V homozygosity, require IL-1i treatment despite the overall good efficacy and tolerability of colchicine, primarily due to colchicine resistance, intolerance, or complications such as amyloidosis.

## 1. Introduction

Familial Mediterranean Fever (FMF) is the most common monogenic autoinflammatory disease and, if left untreated, can lead to a significant disease burden and comorbidities, especially in endemic regions [1,2,3]. Recurrent attacks and inflammation, besides having a negative impact on quality of life, increase the risk of secondary serum amyloid A (AA)-amyloidosis, which is the main cause of long-term morbidity and mortality in FMF patients. According to the most recent European League Against Rheumatology (EULAR) guidelines, the foremost goal of the treatment should be ensuring a proper life quality by controlling attacks, curtailing subclinical and chronic inflammation, and preventing disease-associated complications [4].

Colchicine has been the backbone of FMF treatment since the 1970s, owing to its preventive effects on attacks and secondary amyloidosis [5,6,7]. Despite the overall good safety and tolerability, the use of colchicine is limited due to adverse events, which are estimated to be around 2–5% of all cases [8,9]. Gastrointestinal (GI) side effects, particularly diarrhea, are the most common adverse events and may occur even within the range of recommended colchicine doses. Furthermore, a subset of up to 5% of FMF patients continue to have attacks or subclinical inflammation despite colchicine therapy [8,10,11].

Recently, EULAR proposed a definition for “colchicine resistance” as patients experiencing more than one attack per month despite the maximally tolerated dose of prophylactic colchicine [4]. The development and employment of interleukin-1 inhibitors (IL-1i) heralded a new era in the therapy of FMF, and conferred substantial benefits in controlling disease activity in this challenging subset of patients [12]. IL-1i, namely anakinra, canakinumab, and rilonacept, seem to be promising second-line treatments not only in colchicine-resistant patients, but also in cases of secondary renal AA amyloidosis [12,13]. Despite the efficacy of IL-1inhibiors, several issues limit their use such as cost, accessibility, and potential side effects. Anaphylactic reactions and cytopenia are observed with anakinra, whereas respiratory infections and headache are more frequent with canakinumab. Rilonacept could cause injection site reactions, and is not available in Europe [14,15].

The accruing evidence about the efficacy of anti-IL-1 agents in FMF patients led to their widespread off-label use. Although several risk factors for severe disease course have been identified [16], predicting, which individuals are going to require anti-IL-1 treatment is still beyond reach Therefore, identifying precise indications and clinical characteristics of IL-1i warranting patients are important.

This study aims to evaluate the clinical indications for IL-1 inhibitors and define the patient characteristics in a large cohort of FMF patients at a tertiary care center.

## 2. Materials and Methods

### 2.1. Patient Selection

Medical files of FMF patients aged 18 years and above within the screening period, who were followed up regularly in the rheumatology department of the University of Health Sciences, Basaksehir Cam and Sakura City Hospital, between June 2020 and March 2023 were reviewed retrospectively. Patients who met the criteria for the diagnosis of FMF according to Tel-Hashomer criteria and the New Eurofever/PRINTO classification criteria were included [17]. The following information was obtained from the medical files: demographic data, clinical manifestations of FMF, family history of FMF, genetic testing, histological diagnosis of amyloidosis, and IL1-itreatment. Data were collected from the first visit at which IL1-i was prescribed. The conduction of this study was approved by the ethics committee of the University of Health Sciences, Basaksehir Cam and Sakura City Hospital, Istanbul. (Basaksehir Cam and Sakura City Hospital Local Ethical Committee, Approval Code (protocol number): 2023.12.670, Approval Date: 27 December 2023).

### 2.2. Clinical and Genetic Information

Patient charts were reviewed for clinical manifestations of FMF throughout the whole medical history, including abdominal pain, fever, arthritis, arthralgia, chest pain, and erysipelas-like erythema (ELE). Dose and type of colchicine (i.e., coated or compressed colchicine tablets), colchicine adherence, and observed side effects, namely diarrhea, myelosuppression, and hepatotoxicity, were also collected from the medical records. Colchicine adherence was assessed with patient declaration of regular use. Results of gene analysis were extracted from the files of each patient.

MEFV gene mutations are screened in exons 2, 3, 5, and 10 by polymerase chain reaction (PCR) restriction fragment length polymorphism (PCR-RFLP) technique. Whole venous blood samples treated with ethylenediaminetetraacetic acid (EDTA) were used to extract genomic DNA. PCR was performed in a total volume of 50 µL containing 50 ng of genomic DNA, 5 pmol of each primer, 0.2 mMdNTPs, 1 µL of 10× Fermentas reaction buffer (St. Leon-Rot, Germany), 2 mM magnesium chloride, and 1 unit of Taq DNA polymerase. The amplification conditions were as follows: initial denaturation at 94 °C for 5 min; 30 cycles of denaturation at 94 °C for 1 min; annealing at 65 °C for 1 min; and extension at 72 °C for 1 min; followed by a final extension at 72 °C for 10 min. In order to detect mutations, the restriction endonucleases (Fermentas) were used to digest the PCR products. The digested PCR fragments were separated using electrophoresis on a 2% agarose gel, stained with ethidium bromide, and visualized under ultraviolet light to ascertain the genotypes.

### 2.3. Definition of Colchicine Resistance and Colchicine Intolerance

A complete blood count, liver, and kidney function tests were conducted every three months to detect any potential side effects of colchicine. Subjects were also questioned at each visit for abdominal pain, cramping, hyperperistalsis, diarrhea, nausea, and symptoms of neuromyopathy. Colchicine dose was reduced in a stepwise manner in case of a colchicine-related side effect, such as cytopenia or elevated liver enzymes. Dietary changes or anti-diarrheal/spasmolytic medications were also advised for persistent gastrointestinal adverse effects. Additionally, since several studies have suggested that changing the type of colchicine tablet could help to improve intolerance and lessen side effects [18,19,20], the form of colchicine tablet is changed once before starting anti-IL-1 in the routine clinical practice of the study center. In such circumstances, coated colchicine tablets (Colchicum-Dispert^®^ [Recordati Ilac Sanayi, Istanbul, Turkey] and Kolsin^®^ [Ibrahim Etem Ulagay Ilac Sanayi, Istanbul, Turkey]) were switched to compressed colchicine preparations (Colchicine Opocalcium^®^ [Laboratories Mayoly Spindler, Chatou, France], Colchicine Lirca^®^[Acarpia Farmaceutici S.R.L, Milano, Italy], and Colchicine Seid^®^[Seid S.A., Barcelona, Spain]) with the approval of the Turkish Pharmacists Association. All patients who remained intolerant to the lowest dose of prophylactic colchicine despite all above-mentioned measures were defined as colchicine-intolerant. On the other hand, colchicine resistance was defined as one or more attacks per month, despite the regular use of colchicine at the maximal tolerated dose for at least 6 months [4].

At the time of initiating IL-1i, all patients were receiving at least 2 mg/d colchicine, except those with chronic renal disease and the colchicine intolerance. According to national health regulations in Turkey, FMF patients must have an inadequate response or intolerance to colchicine treatment in order to get a reimbursed IL-1i [21]. In addition, anakinra is used as the first-line IL1-i, followed by canakinumab in the occurrence of unfavorable side effects or inadequate response to anakinra. Anakinra and canakinumab are administered subcutaneously at dose of 100 mg/day and 150 mg/day, respectively. Rilonacept is not available in the country.

### 2.4. Statistical Analysis

Patient characteristics were described using means and standard deviations for continuous variables, as these were not skewed based on histograms and Q-Q plots, counts, and percentages for categorical variables. Welch’s *t*-test was conducted to evaluate the statistical significance of the observed differences between patients requiring biological treatment and those receiving colchicine alone. The Fisher’s exact test was used to compare the frequencies of various homozygous, heterozygous, and compound mutations among patients treated with IL-1i and colchicine combination therapy or colchicine alone. Relative risks (RR) and their corresponding confidence intervals (CIs) were calculated using the riskratio function from the epitools package in R, applying methods such as Wald, small sample, and bootstrap with 5000 replicates to ensure robust estimates. The chi-square test was utilized to assess associations between mutations and treatment types. All *p*-values were two-sided and were considered significant if they were less than 0.05 without multiplicity adjustment. Statistical analyses were conducted using R version 4.3.0 (The R Foundation for Statistical Computing, 2023), employing the readxl, tidyverse, janitor, kableExtra, rstatix, epitools, and sjPlot packages.

## 3. Results

### 3.1. Patient Characteristics

**Figure 1:**
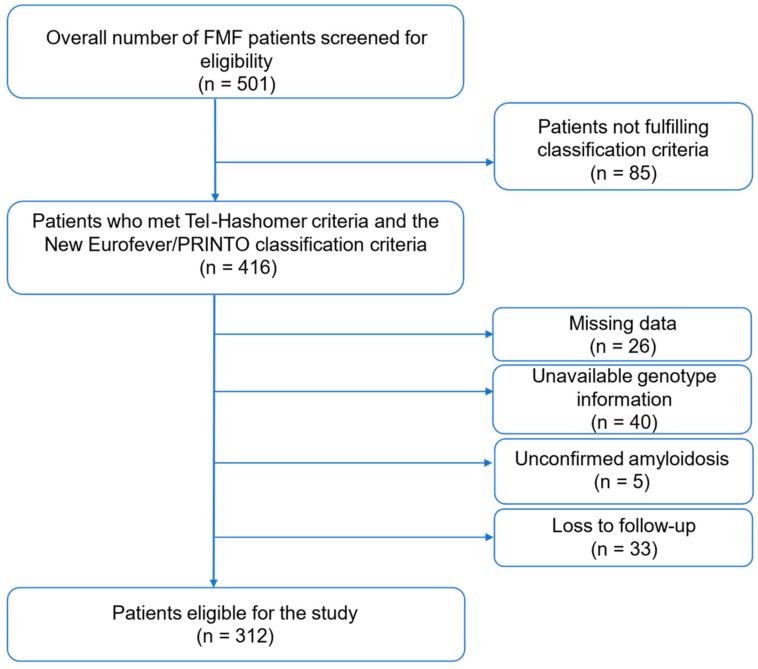
Patient selection flow chart.

An amount of 189 out of a total of 501 screened patients were not included in the study due to not fulfilling the classification criteria, loss to follow-up, missing clinical data, or unavailability of genotype information or unconfirmed amyloidosis (Figure 1). Of 312 study participants, 63% (*n* = 196) were women and 36.8% (*n* = 115) were men. The mean age (standard deviation (SD)) of the population was 34 (11.7). The mean age of disease onset was 14 (9.71) years, whereas the mean age at diagnosis was 26 (12.9) years. Sixty-one percent (*n* = 193) of the patients had a family history of FMF, and 6.7% (*n* = 21) had a family history of amyloidosis.

In total, 87% percent of (*n* = 273)) patients were compliant with colchicine therapy alone or in combination with an IL-1i, while 12% of (*n* = 39) patients were not fully compliant. All but 1% of (*n* = 3) patients were in remission. The most frequent symptom of FMF was abdominal pain, followed by fever and arthralgia, whereas erysipelas like erythema and pericarditis were least frequent. Detailed demographic and clinical characteristics are shown in Table 1.

### 3.2. Genetic Analysis

The screened MEFV gene variants, i.e., M694V, M680I, V726A, K695R, R761H, P369S, E148Q, R202Q, and E167D, were detected overall in 86% (*n* = 270) of our patients (Table 1). The most frequently detected mutation was M694V, with a frequency of 53.2% (*n* = 166), of whom 65 participants were homozygotes. Other pathogenic exon 10 mutations were M680I (17.3%, *n* = 54) and V726A (13.7%, *n* = 43). The M694I variant, which represents another hot spot region, was not detected in any patient. The two rare likely-pathogenic R761H and E167D variants were found in one (0.3%) and two patients (0.6%), respectively [22]. Other encountered MEFV mutations were K365R (0.3%, *n* = 1), P369S (0.6%, *n* = 2), E148Q (14.1%, *n* = 44), and R202Q (22.1%, *n* = 69) (Table 1).

Of the 312 genetic-tested FMF patients fulfilling the diagnostic criteria, 13.4% of them (*n* = 42) lacked any mutations at the screened coding regions.

### 3.3. Indications for IL-1i Treatment

Before initiation of IL-1i, all patients in the cohort were receiving colchicine treatment at a dose of 2 mg/day, except the colchicine-intolerant group and those with renal failure. Eighty-seven percent (*n* = 272) of patients were under colchicine therapy alone. One percent of patients (*n* = 3) failed to achieve remission due to drug non-compliance. Eleven percent (*n* = 37) of patients required treatment with IL-1i, such as anakinra or canakinumab (Table 2).

The most common reason for adding an IL-1i was colchicine resistance (8%, *n* = 25). The presence of AA amyloidosis was the second most common reason (5.1%, *n* = 16). Four percent (*n* = 11) of those patients had colchicine resistance and amyloidosis concomitantly. Despite having achieved clinical disease remission, 1.6% of (*n* = 5) patients required IL-1i treatment due to the development of amyloidosis.

At the beginning of full-dose colchicine therapy, 50 patients experienced untoward effects of colchicine, including diarrhea (*n* = 36), hepatotoxicity (*n* = 12), and cytopenia (*n* = 2). These side effects were alleviated in 35 of patients, either using dose reduction or dietary changes. In the remaining fifteen patients for whom these measures were not successful, coated colchicine tablets were switched to foreign-produced compressed colchicine tablets in eight patients, which subsequently led to the disappearance of side effects and the achievement of disease control in eight patients, while gastrointestinal side effects persisted in seven patients and IL-1i treatment was needed. As such, the prevalence of colchicine intolerance amounted to 2% (*n* = 7) of the cohort and was the third most common reason for IL-1i initiation.

### 3.4. Comparing the Clinical and Genetic Profiles of Patients Who Required IL-1i with Those Who Did Not

**Figure 2:**
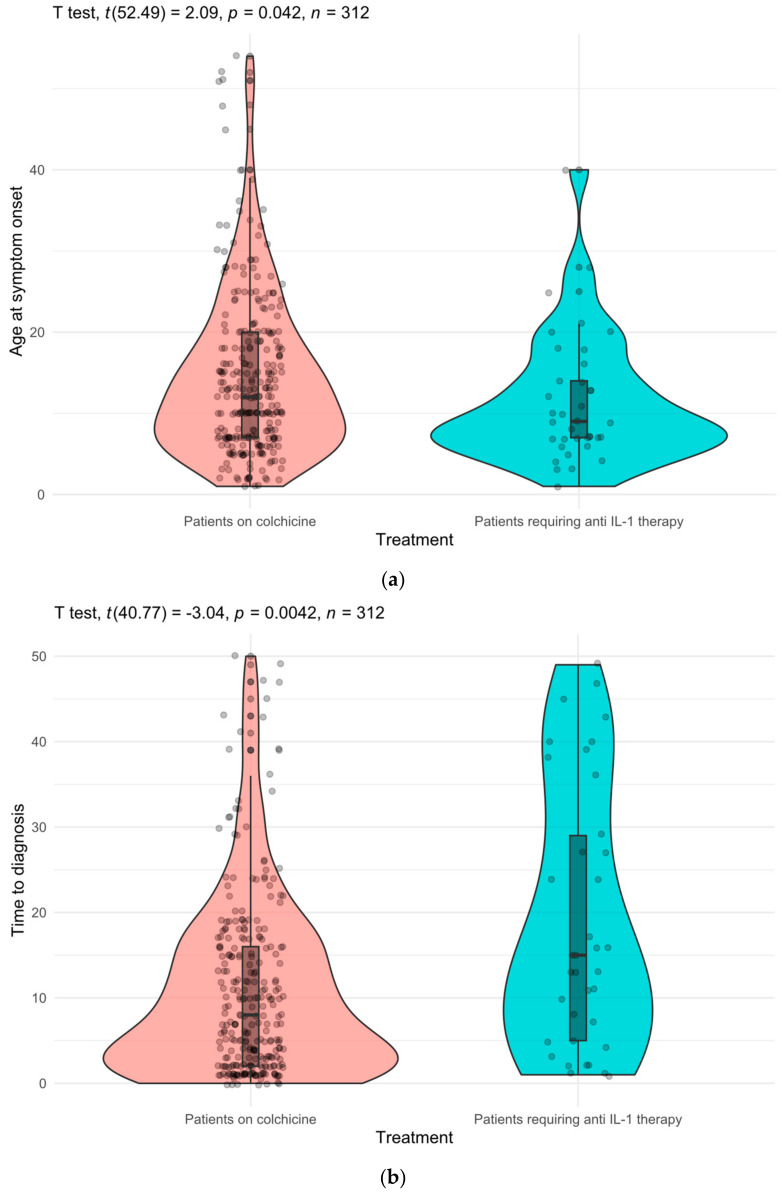
Comparison of biological treatment requiring group with colchicine monotherapy. (**a**) Age at symptom onset. (**b**) Time to diagnosis.

A Welch two-sample *t*-test revealed a statistically significant difference, indicating that the mean age at symptom onset in patients who required IL-1i treatment was lower (11.5 ± 8) compared to patients who did not need IL-1i treatment (14.5 ± 10), (*p* = 0.042) (Figure 2a). We also observed a longer delay from symptom onset to diagnosis in patients requiring IL-1i treatment (18.9 ± 15.4) compared to patients who did not (11 ± 10.6), (*p* = 0.004) (Figure 2b). The frequencies of disease manifestations did not show significant differences between the two groups.

The Fisher’s exact test suggests that there is a statistically significant association between the “Homozygous M694V mutation” and biologic treatment (*p* = 0.001), and the Risk Ratio of 2.90 (95% confidence interval, 1.60 to 5.22) indicates that individuals requiring IL1i treatment were more likely to harbor this genotype in comparison to patients who did not require IL1i treatment. However, we did not find a significant association between biologic treatment and the M694V heterozygote variant or other compound heterozygosity of exon 10 mutations (Table 3).

## 4. Discussion

This single-center, retrospective real-world study aimed to identify the clinical and genetic characteristics of FMF patients requiring IL-1i and the principal rationales for this treatment. To the best of our knowledge, this is the largest cohort of FMF patients mainly focusing on indications of IL-1i therapy. In terms of clinical aspects, disease presentations did not differ between the IL-1i treated group and the colchicine monotherapy group. However, the age of symptom onset was younger, and the time to diagnosis was longer in the IL-1i-treated group, with an average diagnostic delay of nearly 20 years, which likely contributes to poorer quality of life and more frequent disease complications. Genetically, M694V homozygosity was associated with an increased biological treatment requirement. This is consistent with previous studies, which showed that the M694V/M694V genotype is associated with a more serious disease phenotype with earlier age of symptom onset, more frequent FMF attacks, and more frequent resort to IL-1i treatment [23,24,25].

Considering patient characteristics using IL-1i therapy, M694V homozygosity was associated with an increased biological treatment requirement. This is consistent with previous studies, which showed that the M694V/M694V genotype is associated with a more serious disease phenotype with earlier age of symptom onset, more frequent FMF attacks, and more frequent resorting to anti-IL-1 treatment [26,27,28].

In general, the most frequent indication of IL-1i therapy was (i) colchicine resistance, followed by (ii) secondary amyloidosis and (iii) colchicine intolerance. Notably, colchicine resistance was accompanied by amyloidosis in a conspicuous subgroup of patients, likely reflecting the higher overall disease activity in this subset. Over the past decade, the concept of “colchicine resistance” has undergone many modifications, and a definite consensus is yet to be reached [29]. Previous reports have shown that about 5–10% of FMF patients fail to achieve adequate disease control with colchicine monotherapy [7,12,29,30]. Despite a respectable proportion of fully compliant patients, however, the frequency of colchicine resistance was higher in our cohort [7,8,30]. A plausible explanation for that might be more than half of the patients had the mutant M694V allele, which is mainly associated with worse disease control and outcomes [2,24,25], whereas another plausible explanation could be misreported treatment compliance with colchicine.

The frequency of amyloidosis was 5% in the current study, in line with previous studies [21,29,31,32]. Thanks to colchicine and IL-1i, the incidence of amyloidosis decreased incrementally [25,31,33]; nonetheless, thus far, there is no definitive cure for it. Despite the proven preventive effects of colchicine on amyloidosis [6,34,35,36], its role in the treatment of amyloidosis is not yet defined. On the other hand, the preventive effect of IL-1i on amyloidosis is not yet established, whereas several observations suggest that these drugs could be effective for the treatment of AA amyloidosis, supposedly by inhibition of excessive amyloid fibril deposition and amyloid fibril toxicity [21,37]. Based on the available evidence, current guidelines recommend initiating IL-1i treatment in cases of secondary AA-amyloidosis in FMF, regardless of disease activity [4].

Colchicine side effects were an important reason for adding biological treatment despite precautions including dose reduction, switching to compressed tablets, and dietary changes. The main contributors to colchicine intolerance were GI side effects. GI symptoms are dose-dependent, and are associated with decreased disaccharidase activity in the jejunum [38]. Adverse GI effects could be ameliorated by dose reduction or lowering dietary lactose intake [39,40]. In addition, drug formulation might have an impact on intolerance as well as resistance. Several studies from Turkey pointed out that switching coated colchicine preparations to compressed forms could yield additional benefits on disease control and drug intolerance [18,19,20]. Notwithstanding the lack of concrete pharmacokinetic evidence, it seems like embracing an akin approach in daily clinical practice helped us to improve drug tolerance. Hepatotoxicity and cytopenia were relatively rare side effects which resolved after reducing the dose of colchicine and did not require further treatment.

The focus of FMF treatment is repressing autoinflammation, thereby avoiding its detrimental consequences. According to the recent EULAR guidelines, along with the prevention of attacks and potential disease complications, treatment of FMF should also focus on abolishing chronic subclinical inflammation [41]. Correspondingly, the current recommendations suggest increasing the colchicine dose in the presence of ongoing subclinical inflammation during the attack-free period. However, there is still no clear recommendation regarding the use of IL-1i in patients who continue to have high inflammatory parameters even after increasing the colchicine dose [4]. Early identification those FMF patients with uncontrolled inflammation is crucial, as it could lead to disease-related consequences and irreversible damage [32]. In our study, only a small proportion of patients had persistently elevated inflammation parameters, and were accompanied by either colchicine resistance or amyloidosis; thus, they still received IL-1i therapy.

Our study has several limitations. Due to the nature of the retrospective study design, our analysis did not include follow-up data on disease activity measures such as the autoinflammatory disease activity index or FMF-50 index. Therefore, no quantitative observations on disease burden or standardized quality of life assessments (i.e., SF-36) could be made regarding the effectiveness of colchicine, IL-1i, or comparison of IL1-i effectivity (anakinra vs. canakinumab) in our cohort. Moreover, the colchicine monotherapy group might also include patients with active disease, who might need IL-1i treatment in the future. Lastly, the age of symptom onset was based on patient-reported data, which could cause measurement errors, especially in patients with long disease duration.

## 5. Conclusions

Data from this large real-life cohort of FMF show that colchicine is generally well-tolerated and effective in preventing recurrent attacks and amyloidosis in most patients. However, a relevant subset of patients will still require IL-1i therapy due to colchicine resistance, intolerance, or if secondary amyloidosis is developed. In all cases, patients receiving IL-1i usually have long-standing disease and/or severe genetic endotypes such as the M694V mutation, and thus bear higher chances for complications. Therefore, it is pivotal to identify patients who cannot be adequately treated with colchicine early to prevent side effects, loss of quality of life, and FMF complications. Defining this subset of patients would help to initiate IL-1i treatment without delay in a tailored and cost-effective manner. To achieve this, larger prospective studies are needed to better define disease and therapy outcomes, as well as the establishment of registries for pooling clinical and genetic information.

## Figures and Tables

**Table 1:** Baseline participant characteristics.

			Overall	Patients on Colchicine Monotherapy	Patients Requiring IL-1i
Number of participants, *n*			312	275	37
Age, mean (SD)			34 (11.7)	33.5 (11.4)	40.5 (12.6)
Male/Female, *n* (%)			115/196 (36.8/63.2)	101/173 (36.7/62.9)	14/23 (4.4/7.3)
Age at symptom onset, years, mean (SD)			14.0 (9.71)	14.5 (9.9)	11.5 (7.97)
Age at diagnosis, years, mean (SD)			26.0 (12.9)	25.5 (12.3)	30.4 (16.3)
Time between symptom onset and diagnosis, years, mean (SD)			11.9 (11.6)	11.0 (10.7)	18.2 (15.6)
Family history of FMF, *n* (%)			193 (61.8)	170 (61.8)	23 (62.1)
Family history of amyloidosis, *n* (%)			21 (6.7)	17 (6.1)	4 (10.8)
Symptoms/manifestations *n* (%)	Abdominal pain		292 (93.5)	259 (94.1)	33 (89.1)
	Fever		246 (78.8)	215 (78.1)	31 (83.7)
	Arthralgia		180 (57.6)	159 (57.8)	21 (56.7)
	Arthritis		122 (39.1)	105 (38.1)	17 (45.9)
	Pleuritic pain		107 (34.2)	99 (36.0)	8 (21.6)
	Erysipelas-like erythema		67 (21.4)	55 (20.0)	12 (32.4)
	Pericarditis		2 (0.6)	2 (0.7)	0 (0.0)
MEFV variant *n* (%)	Exon 10	M694V	166 (53.2)	143 (52.0)	23 (62.1)
		M680I	54 (17.3)	50 (18.1)	4 (10.8)
		V726A	43 (13.7)	41 (14.9)	2 (5.4)
		M694I	-	-	-
		K695R	1 (0.3)	1 (0.3)	-
		R761H	1 (0.3)	1 (0.3)	-
	Exon 3	P369S	2 (0.6)	1 (0.3)	1 (2.7)
	Exon 2	R202Q	69 (22.1)	62 (22.5)	7 (18.9)
		E148Q	44 (14.1)	39 (14.1)	5 (13.5)
		E167D	2 (0.6)	2 (0.7)	-

IL-1i: interleukin-1 inhibitor, FMF: familial Mediterranean fever, MEFV: Mediterranean fever.

**Table 2:** Distribution of patients according to treatment groups and compliance.

	Patients on colchicine monotherapy		272 (87.1%)
Overall cohort (*n* = 312)	Patients requiring IL-1i *		37 (11.8%)
	-	Colchicine resistance	25 (8.0%)
	-	Amyloidosis	16 (5.1%)
	-	Colchicine intolerance	7 (2.2%)
	Non-compliant patients		3 (0.9%)

Data are presented as absolute number (*n*) with proportion (%). IL-1i: interleukin-1 inhibitor. *: Subcategories are not mutually exclusive.

**Table 3:** Allele frequencies of FMF mutations according to the treatment groups.

	Patients on Colchicine Monotherapy (*n* = 275)	Patients Requiring IL-1i (*n* = 37)	RR (95% CI)	*p* Value
Homozygous M694V	49 (17.8%)	16 (43.2%)	2.90 (1.60–5.22)	<0.001
M694V mutation (homozygote, single or with other MEFV variants)	143 (52.0%)	23 (62.2%)	1.44 (0.77–2.70)	0.294
M694V/V726A compound heterozygotes	15 (5.5%)	1 (2.7%)	0.51 (0.08–3.51)	0.704
M694V/M680I compound heterozygotes	18 (6.5%)	2 (5.4%)	0.83 (0.22–3.22)	>0.999 *
M680I/V726A compound heterozygotes	2 (0.7%)	0 (0%)	- ^§^	>0.999 *

Data are presented as absolute number (*n*) with proportion (%). IL-1i: interleukin-1 inhibitor, MEFV: Mediterranean fever. *: This result is caused by the fact that the observed values in each cell of the contingency table are the same as the expected values. ^§^: It is not possible to calculate the risk ratio because the number of patients taking biologics with compound mutation of M680I and V726A is zero.

## Data Availability

Data from this study can be requested from the corresponding author upon reasonable request.
